# Modulation Spectra Capture EEG Responses to Speech Signals and Drive Distinct Temporal Response Functions

**DOI:** 10.1523/ENEURO.0399-20.2020

**Published:** 2021-01-05

**Authors:** Xiangbin Teng, Qinglin Meng, David Poeppel

**Affiliations:** 1 Department of Neuroscience, Max-Planck-Institute for Empirical Aesthetics, Frankfurt 60322, Germany; 2 Acoustic Laboratory, School of Physics and Optoelectronics, South China University of Technology, Guangzhou 510641, China; 3 Department of Psychology, New York University, New York, NY 10003; 4 Max-Planck-NYU Center for Language, Music, and Emotion, New York University, New York, NY 10003

**Keywords:** amplitude envelope, auditory receptive field, neural entrainment, speech perception, temporal processing, temporal window

## Abstract

Speech signals have a unique shape of long-term modulation spectrum that is distinct from environmental noise, music, and non-speech vocalizations. Does the human auditory system adapt to the speech long-term modulation spectrum and efficiently extract critical information from speech signals? To answer this question, we tested whether neural responses to speech signals can be captured by specific modulation spectra of non-speech acoustic stimuli. We generated amplitude modulated (AM) noise with the speech modulation spectrum and 1/f modulation spectra of different exponents to imitate temporal dynamics of different natural sounds. We presented these AM stimuli and a 10-min piece of natural speech to 19 human participants undergoing electroencephalography (EEG) recording. We derived temporal response functions (TRFs) to the AM stimuli of different spectrum shapes and found distinct neural dynamics for each type of TRFs. We then used the TRFs of AM stimuli to predict neural responses to the speech signals, and found that (1) the TRFs of AM modulation spectra of exponents 1, 1.5, and 2 preferably captured EEG responses to speech signals in the δ band and (2) the θ neural band of speech neural responses can be captured by the AM stimuli of an exponent of 0.75. Our results suggest that the human auditory system shows specificity to the long-term modulation spectrum and is equipped with characteristic neural algorithms tailored to extract critical acoustic information from speech signals.

## Significance Statement

Speech signals have a unique long-term modulation spectrum shape that differs speech from other natural sounds. Does the human auditory system adapt to the speech long-term modulation spectrum and efficiently extract critical information from speech signals? To answer this question, we generated artificial sounds with various modulation spectra and examined whether neural encoding models derived from specific modulation spectra can better explain neural responses to speech signals than others. We found that the modulation spectra with the exponents that are close to the speech modulation spectrum preferably captured electroencephalography (EEG) responses to speech signals than others. Our results suggest that the human auditory system shows high sensitivity to the long-term modulation spectrum specific to speech signals.

## Introduction

Sensory systems evolve to adapt to environmental statistics and to efficiently extract features in natural stimuli essential to animals’ survival (Barlow, 1961). For instance, the human brain is equipped with cortical areas and neural processing algorithms specialized for recognizing facial features (Kanwisher et al., 1997; Tsao and Livingstone, 2008). In parallel, speech is fundamental to human communication, and hence the human auditory system must evolve to be sensitive to unique acoustic properties of speech signals (Belin et al., 2000; Overath et al., 2015). One acoustic feature that differentiates speech from other natural sounds is long-term modulation spectrum (Ding et al., 2017). Natural sounds, such as environmental noise, speech, music, and some vocalizations, often have broadband modulation spectra that show a 1/f pattern with its exponent indicating how sounds are modulated across various timescales (Voss and Clarke, 1978; Theunissen and Elie, 2014). Compared with environmental noise and some vocalizations, speech has a unique modulation spectrum with an exponent of frequency between 1 and 1.5 (Singh and Theunissen, 2003) and a prominent peak around 4 Hz (Ding et al., 2017; Varnet et al., 2017). Does the human auditory system show sensitivity to the specific shape of speech long-term modulation spectrum?

Characteristic temporal dynamics of sounds (i.e., temporal autocorrelation and periodicity) manifests in their long-term modulation spectra (Wiener, 1930). Speech is quasi-periodic with syllables ranging from 150 to 300 ms (Rosen, 1992); speech intonations and pitch contours span across multiple syllables and reside in an even larger timescale (>500 ms; Ghitza and Greenberg, 2009). The long-term modulation spectrum of speech can be considered as a first-order summary statistics that characterizes such multiscale temporal dynamics. The slope of the speech modulation spectrum indicates that the first-order acoustic information is primarily carried by the low-frequency range (e.g., <10 Hz). To efficiently process speech signals, the human auditory system may develop canonical neural algorithms (e.g., specialized receptive fields) to extract essential acoustic information over multiple timescales manifested in the speech modulation spectrum (Poeppel, 2003; Ghitza and Greenberg, 2009; Ghitza, 2012; Giraud and Poeppel, 2012). The long-term modulation spectrum of speech signals may prove to be crucial to speech perception, and artificial sounds with a speech-like modulation spectrum may efficiently drive speech-specific neural responses of the human auditory system. Validating those hypotheses can help deepen our understanding of fundamental neural mechanisms of speech perception and potentially reveal speech-specific auditory processes, analogous to face-specific neural processes (Tsao and Livingstone, 2008).

Here, we employed an electroencephalography (EEG) encoding framework to derive temporal response functions (TRF) from speech signals and artificial sounds with modulation spectra typical of speech signals and other natural sounds (Di Liberto et al., 2015; O'Sullivan et al., 2015; Holdgraf et al., 2017). The rationale is that, if the auditory system simply responds to temporal changes in sounds and is indifferent to the shape of long-term modulation spectra of sounds, TRFs derived from sounds with one type of modulation spectrum should be able to generalize across sounds of different shapes of modulation spectra. In contrast, if long-term modulation spectra are indeed critical to different types of natural sounds, the TRFs derived from sounds with different modulation spectra should manifest specificity to the corresponding modulation spectrum. Artificial sounds with a speech-like modulation spectrum, but not other modulation spectra, would drive the auditory system in a similar manner as speech signals, and the TRFs derived from those artificial sounds of the speech-like modulation spectrum should be able to predict the neural responses to speech signals.

We selected a natural speech excerpt and generated amplitude modulated (AM) sounds with a speech modulation spectrum and with 1/f modulation spectra with different exponents (Garcia-Lazaro et al., 2006). In the first session of the experiment, while recording EEG signals, we presented the AM stimuli to participants who were instructed to detect a short tone inserted in half of the AM stimuli. In the second session, the participants listened to the speech excerpt while undergoing EEG recording. We derived TRFs from each type of AM stimuli, which were then used to predict neural responses to the speech material. We were interested to see which TRFs derived from the AM stimuli could best capture neural responses to natural speech. Moreover, we investigated how acoustic information was encoded in different frequency bands of neural signals and tested how different frequency bands of amplitude modulations of sounds contributed to encoding neural signals.

## Materials and Methods

### Participants

Twenty-one native German speakers (age 23–49, one left-handed, eight females) took part in the experiment. All participants had normal hearing and no neurologic deficits according to their self-report. Two participants were excluded because one participant did not finish the experiment and the EEG recordings from the other participant lacked triggers for stimulus onsets. The formal analyses included 19 participants (ages 23–49, one left-handed, eight females). Written informed consent was obtained from each participant before the experiment and monetary compensation was provided after the experiment. The experimental protocol was approved by the Ethics Council of the Max Planck Society.

### Stimuli

We selected a German TEDx talk given by a native male German speaker, Redesigning Design (www.youtube.com/watch?time_continue=784&v=dAljDx_lSQ4), and extracted the audio track using an online tool (which is not available any more. Please contact the corresponding author for information regarding the audio track). The audio data from the 4th minute to the 13th minutes of the talk were further selected to avoid musical contents as well as vocal and clamping sounds from the audience. Therefore, a 10-min recording of German speech material was used in the current experiment. The sampling rate of the speech material was 20,000 Hz, and the amplitude was normalized to 70-dB sound pressure level (SPL) by referring the speech material to a 1-min white noise piece, which was measured beforehand to be 70-dB SPL at the experimental setting.

**Figure 1.**
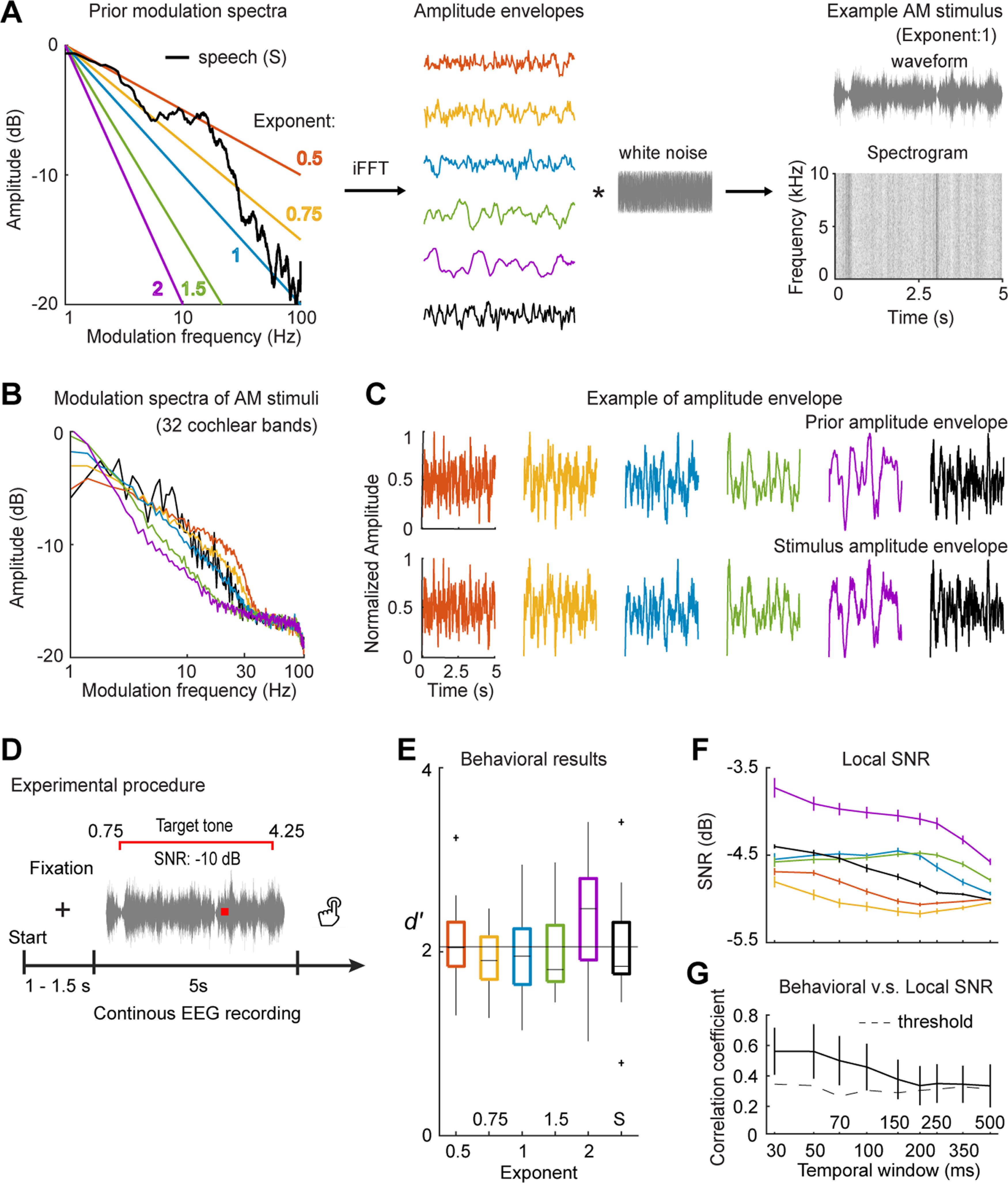
Stimulus generation, experimental paradigm, and behavioral results. ***A***, AM stimulus generation. The left panel shows the modulation spectra used to generate AM envelops. The line color codes for different modulation spectra. An example of each AM envelope is shown in the middle panel. We filtered the AM envelopes through a bandpass filter of 1–30 Hz and then modulated broadband white noise with the AM envelopes to create AM stimuli. An example waveform of the AM stimuli with a 1/f modulation spectrum of exponent 1 is shown in the upper right panel. The spectrogram of the example AM stimulus is shown in the lower right panel. ***B***, Modulation spectra of AM stimuli. We extracted amplitude envelopes of each AM stimulus and then converted the envelopes to modulation spectra for each AM type. It can be seen that the trends of the prior modulation spectra were preserved in the modulation spectra of the AM stimuli. ***C***, Examples of prior AM envelopes and the AM envelopes from the AM stimuli. The upper row shows examples of the prior AM envelopes and the lower row shows the AM envelopes extracted from the AM stimuli. ***D***, Experimental paradigm for presenting AM stimuli during EEG recording. ***E***, Box plot of behavioral data. D-prime values were calculated to quantify the performance of tone detection. The thin black line indicates the threshold of significance (α level of 0.01) derived from a permutation test (for more details, see Results). ***F***, Local SNR of tones in the AM stimuli. We calculated local SNRs using temporal windows of different sizes. The line color codes for different AM types as in ***A***. ***G***, Correlation between behavioral data and local SNR. The dashed line represents the threshold of significance (α level of 0.01) derived from a permutation test (for more details, see Results). The results show that the shorter the temporal window is, the better the local SNR explains the behavioral performance. The error bars in ***F***, ***G*** represent ±1 SE over participants. The AM stimuli can be found in the OSF project folder https://osf.io/yp4k3/.

We followed the methods used in Garcia-Lazaro et al. (2006) to generate AM stimuli with 1/f modulation spectra of different exponents and the modulation spectrum of the speech material. A schematic plot of the stimulus generation process is shown in Figure 1*A*. We first generated AM envelopes of 1/f modulation spectra using an inverse Fourier method. We fit the modulation spectra to have 1/f shapes with exponents at 0.5, 0.75, 1, 1.5, and 2 (Fig. 1*A*, left panel) and converted the spectra from the frequency domain to the temporal domain using inverse fast Fourier transformation (iFFT). The phase spectra were obtained from pseudo-random numbers drawn uniformly from the interval [0, 2π]. We fixed the sampling rate to 20,000 Hz and then created modulation spectra of 20,000 × 10 points with a frequency range of 0–10,000 Hz, so that each generated envelope was 10 s long. Using different random number seeds for the phase spectra, we were able to generate 60 AM envelopes (Fig. 1*A*, middle panel) with different dynamics (modulation phase) for each exponent. We next applied the same procedure to generate AM stimuli with the modulation spectrum of the speech material. We divided the speech material into ten 1-min pieces and used one frequency band covering the frequency range between 80 and 8000 Hz to extract the broadband AM envelope from each 1-min piece. The speech AM envelopes were converted to modulation spectra using FFT and were then averaged across the ten speech pieces. We downsampled the modulation spectrum so that the averaged speech modulation spectrum had 20,000 × 10 points, from which we generated 60 AM envelopes of 10 s following the same procedure generating the 1/f AM envelopes.

All the AM envelopes generated from 1/f modulation spectra and speech modulation spectra were then filtered with a high-pass Butterworth filter of an order 3 at 1 Hz and a lowpass Butterworth filter of an order 6 at 30 Hz. We selected envelope segments of 5 s from the middle of the 10-s envelopes for further usage to avoid artifacts caused by filtering in the beginning and the end of the filtered AM envelopes. All the selected AM envelopes (5 s long) were normalized to have a modulation depth of 100%, which is that the largest point of the envelopes had a magnitude of 1 and the lowest point had a magnitude of 0.

The AM stimuli were generated by modulating broadband white noise with the AM envelopes created above. We first generated a 5 s piece of white noise using a random number generation function, ‘randn,’ in MATLAB R2016b (The MathWorks) at a sampling rate of 20,000 Hz and then directly modulated the amplitude of the noise piece using the AM envelopes without dividing the noise piece into different frequency bands. Each piece of white noise was independently generated for each AM stimulus. We generated 60 AM stimuli with different modulation phases for each type of AM envelopes, so that each AM stimulus of each type of AM envelopes had distinct modulation phases from the other 59 AM stimuli. Hence, we had six modulation spectra (five types of 1/f modulation spectra and one speech modulation spectrum) and totally 60 × 6 AM stimuli. We applied a cosine ramp-up function in a window of 50 ms at the onset of all AM stimuli and a sine ramp-down function of 50 ms at the offset. The amplitude of the AM stimuli was normalized to ∼70-dB SPL.

All the AM stimuli and scripts for generating materials and analyses can be found in the OSF project folder of the present study: https://osf.io/yp4k3/.

### Acoustic analysis on stimuli

To characterize amplitude modulations of the AM stimuli and to simulate outputs of cochlear filters, we computed an averaged modulation spectrum for each type of AM stimuli using a gammatone filterbank (Fig. 1*B*). We filtered the AM stimuli through a gammatone filterbank of 32 bands logarithmically spanning from 80 to 8000 Hz (Patterson et al., 1987; Ellis, 2009). The envelope of each cochlear band was extracted by applying Hilbert transformation on each band and taking the absolute values (Glasberg and Moore, 1990; Søndergaard and Majdak, 2013). The amplitude envelopes across 32 bands were then averaged and transformed to a modulation spectrum using FFT. We averaged the modulation spectra across the 60 AM stimuli for each type of AM envelopes. The a posteriori AM spectra preserved the shape of modulation spectra defined a priori (Fig. 1*A*) and the comparisons of AM envelopes in the temporal domain also demonstrated preserved similarity between the a priori and a posteriori AM envelopes (Fig. 1*C*).

### Experimental protocol and EEG recording

EEG data were recorded using an actiCAP 64-channel, active electrode set (10–20 system, Brain Vision Recorder, Brain Products), at a sampling rate of 500 kHz, with a 0.1-Hz online filter (12 dB/octave roll-off). There were 62 scalp electrodes, one electrode (originally, Oz) was placed on the tip of the nose. All impedances were kept below 5 kΩ, except for the nose electrode, which was kept below ∼10 kΩ.

The experiment included two sessions. In the first session, all the AM stimuli were presented in a randomized order to each participant during EEG recording. A 1000-Hz pure tone of 30-ms duration was randomly inserted into the half of the AM stimuli (30 stimuli for each type of AM envelopes), and the onset of the tone was randomly distributed between 0.75 and 4.25 s (Fig. 1*D*). The signal-to-noise (SNR) of the tone to the AM stimuli was fixed at −10 dB, because in the preliminary test we determined that a tone at a SNR of −10 dB could be detected at an adequate rate (i.e., avoiding ceiling or floor effects). We applied a cosine ramp-up function in a window of 10 ms at the onset of the tone and a sine ramp-down function of 10 ms at the offset. After each AM stimulus was presented, the participants were required to push one of two buttons to indicate whether they heard a tone in the AM stimulus. Between 1 and 1.5 s after participants responded, the next stimulus was presented. The AM stimuli were presented in four separate blocks with 90 trials in each block. After each block, the participants could choose to take a short break or to start the next block. An illustration of the experimental procedure in this session can be seen in Figure 1*D*.

In the second session, participants were presented with the speech material while undergoing EEG recording and were required to summarize the contents of the speech material after the recording. The behavioral task was designed to maintain participants’ focus on the speech material and hence the participants’ summaries of the speech material were not recorded or analyzed.

During the stimulus presentation in the both sessions, participants were required to keep eyes open and to fix on a white cross in the center of a black screen. The auditory stimuli were delivered through plastic air tubes connected to foam ear pieces (E-A-R Tone Gold 3A Insert earphones, Aearo Technologies Auditory Systems).

### Behavioral data analysis

Behavioral data were analyzed in MATLAB 2016b (The MathWorks; RRID:SCR_001622) using the Palamedes toolbox 1.5.1 (RRID:SCR_006521; Prins and Kingdom, 2009). For each AM envelope type, there were 60 stimuli, half of which had a tone embedded. A two-by-two confusion matrix was created for each AM envelope type by treating the trials with the tone embedded as “target” and the other trials as “noise.” Correct detection of the tone in the target trials was counted as “hit,” while reports of hearing a tone in the noise trials were counted as “false alarm”; D-prime values were computed based on hit rates and false alarm rates of each table. A half artificial incorrect trial was added to the table with all correct trials (Macmillan and Creelman, 2004).

### Local SNR of the embedded tones

The modulation spectra of AM stimuli led to different temporal dynamics and modulated local SNRs of the embedded tones. The differences of local SNR between AM envelope types could potentially explain the behavioral performance of tone detection. Therefore, we calculated the local SNR of the embedded tones using rectangular temporal windows of different sizes. We did not vary the frequency bandwidth within the temporal windows but calculated power within each temporal window in the temporal domain, because the AM stimuli were generated by modulating broadband white noise without decomposing white noise into different frequency bands and each frequency range can be considered to be equally modulated. We chose nine temporal window sizes: 30, 50, 70, 100, 150, 200, 250, 350, and 500 ms and centered the temporal window in the middle of the tone, 15 ms after tone onset, and computed power of the AM stimuli without the tone in this temporal window. Then, to compute local SNR, we divided the power of the tone by the power of the AM stimuli within the temporal window. We transformed the values of local SNR into decibels by taking a log with base 10 and multiplying by 10.

### EEG preprocessing and analysis

EEG data analysis was conducted in MATLAB 2016b using the Fieldtrip toolbox 20181024 (RRID:SCR_004849; Oostenveld et al., 2011) and the wavelet toolbox in MATLAB. EEG recordings were off-line referenced to the average of activity at all electrodes. Raw EEG data were first filtered through a bandpass filter from 1 to 45 Hz embedded in the Fieldtrip toolbox (a FIR zero-phase forward and reverse filter using MATLAB ‘fri1’ function with an order of 4). Trials were then visually inspected, and those with artifacts such as channel jumps and large fluctuations were discarded. An independent component analysis was applied separately for EEG recording of each experimental session and used to correct for artifacts caused by eye blinks and eye movements. After preprocessing, up to 10 trials were removed for each AM type. To avoid biased estimation in the following analyses, we only included 50 trials in the analyses for each AM envelope type (∼83% of data). Each trial was divided into a 11-s epoch, with a 3-s prestimulus period and a 3-s poststimulus period. Baseline was corrected for each trial by subtracting out the mean of −1 to 0 s in each trial.

#### Auditory component extraction

**Figure 2.**
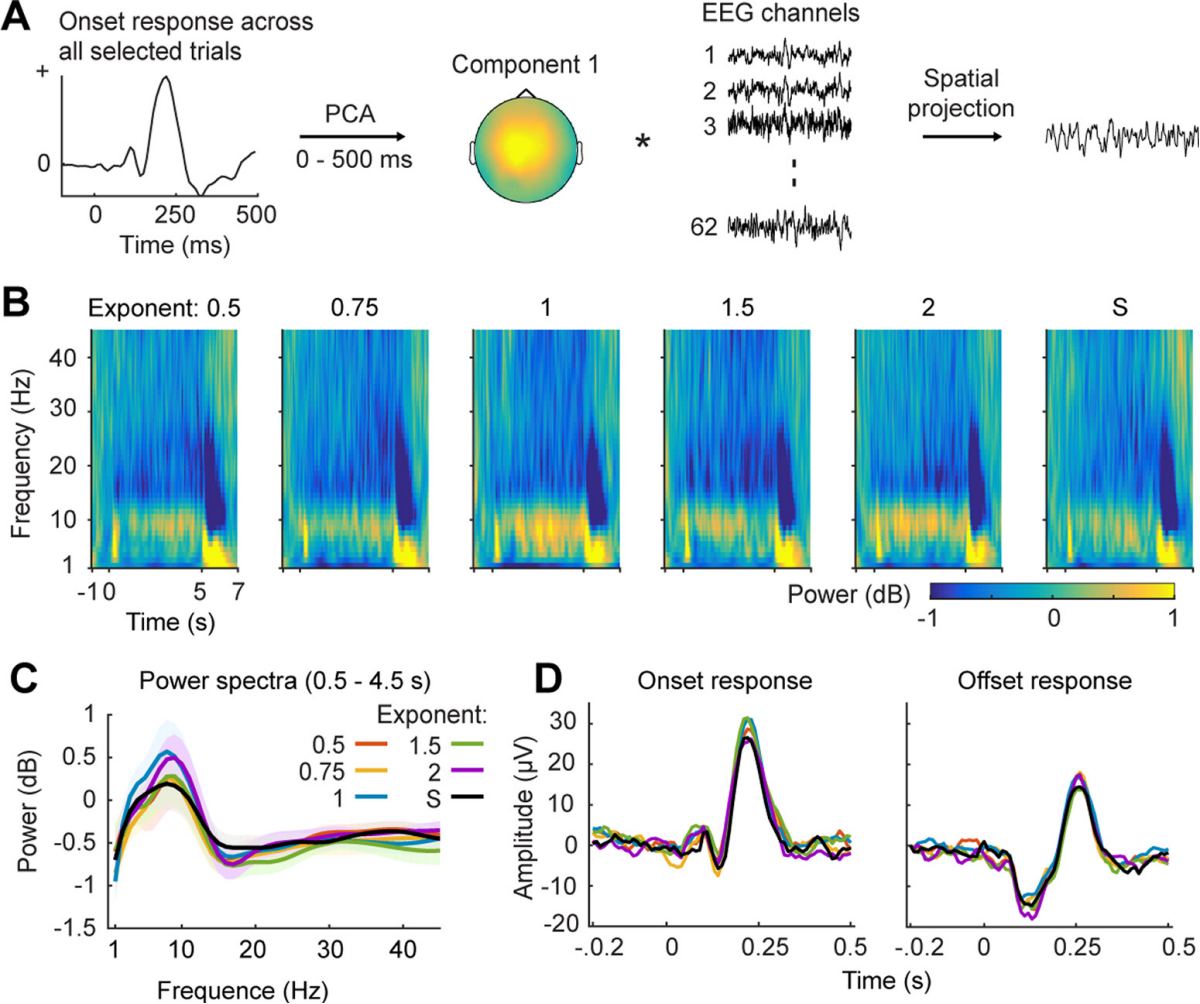
Spatial projection of EEG signals, induced power, and onset/offset responses. ***A***, PCA component extraction and EEG spatial projection. To extract auditory responses across different EEG electrodes, we averaged EEG signals across all the selected trials and calculated the onset response to the stimulus onset (left panel). Five PCA components were extracted across all EEG channels and the first PCA component, which explained the largest variance, was selected. The middle panel shows an example topography of weights of the first PCA component from one participant. We then projected EEG signals of each trial across electrodes to the first PCA component using its weighting matrix and derived signals that summarized auditory-related responses across EEG electrodes. ***B***, Spectrograms of induced power for each type of AM stimuli. From left to right, each spectrogram represents induced power of each AM type. ***C***, Induced power spectra. We averaged induced power from 0.5 to 4.5 s after stimulus onset for each type of AM stimuli to avoid influences of onset and offset responses and motor components caused by button presses. The line color codes for different AM spectra as in Figure 1. The shaded area represents ±1 SE over participants. No significant differences were found between different AM types (*p *>* *0.05). ***D***, Onset and offset responses to each type of AM stimuli. The line color codes for different AM type. No significant differences were found between different AM types (*p *>* *0.05).

We primarily focused on neurophysiological signals evoked by auditory stimuli in this study. To extract EEG signals mostly reflecting sound-related responses instead of arbitrarily selecting certain electrodes (e.g., CZ or FCZ), we derived a spatial filter using principal component analysis (PCA; Fig. 2*A*). We first averaged over all the trials of the AM stimuli (300 trials) for each participant and calculated an evoked response to the stimulus onset at each EEG electrode. PCA was then applied on the evoked response from 0 to 500 ms after the stimulus onset across all electrodes. For each participant, we selected the weighting matrix (spatial filter) of the first PCA component and then applied the spatial filter both on each trial of the AM stimuli and on the EEG recording of the speech material, so that the derived signals were weighted over all EEG electrodes and reflected summarized auditory components. As PCA sometimes reversed polarity of EEG signals, the polarity of the derived signals was manually checked and corrected for each participant. This procedure of component extraction simplified further analyses and avoided biases introduced by differences of EEG cap positions and head sizes across participants. We conducted all the analyses on the derived signals.

#### Evoked responses to stimulus onset and offset

We calculated evoked responses to the onset and the offset of the AM stimuli for each AM envelope type. Baseline was corrected using the EEG signals between −200 and 0 ms before stimulus onset.

#### Induced power analysis

As each AM stimulus had different dynamics from the other AM stimuli, we chose to calculate induced power but not indices sensitive to congruence of phases across trials, such as inter-trial phase coherence and evoked power. To extract time-frequency information, single-trial data from the derived EEG signals were transformed using functions of Morlet wavelets embedded in the Fieldtrip toolbox, with frequencies ranging from 1 to 45 Hz in steps of 1 Hz. To balance spectral and temporal resolution of time-frequency transformation, window length increased linearly from 2 to 10 cycles from 1 to 45 Hz. Power responses were extracted from the wavelet transform output at each time-frequency point and then were averaged across AM stimuli for each AM type. We normalized the averaged power responses by dividing the mean power value in the baseline range (−1 to −0.75 s) and converted them to decibel units. We calculated induced power spectra by averaging the induced power responses for each AM type from 0.5 to 4.5 s poststimulus to avoid effects of neural responses evoked by stimulus onset and offset.

### EEG encoding analysis

To investigate how the auditory system encodes acoustic dynamics of different modulation spectra, we employed an encoding framework to predict EEG signals using amplitude envelopes of different AM types. The underlying hypothesis is that, if the auditory system tunes to certain shapes of AM spectra and acoustic dynamics of the corresponding AM stimuli efficiently drive auditory responses, the amplitude envelopes of such AM stimuli can be used to predict auditory responses in EEG signals with high accuracy. Moreover, if an AM type drives auditory responses in a similar manner that speech signals drive auditory responses, the kernel trained using this AM type can be also used to predict auditory responses to speech signals. Hence, we could draw a conclusion that the shape of modulation spectrum of this AM type can capture canonical auditory responses to speech signals.

The method used here is to map between amplitude envelopes of AM stimuli averaged across cochlear bands (for details, see Acoustic analysis on stimuli) and the EEG signals. A TRF was derived from the amplitude envelopes of stimuli (*S* with subscript *c* indicating critical band) and their corresponding EEG signals (*R* with subscript *b* indicating neural band) through ridge regression with a parameter (λ) to control for overfitting (superscript *t* indicating transpose operation):

$$TRF_{c,b}={\left(R_{b}^{t}R_{b}+\lambda I\right)}^{-1}R_{b}S_{c}.$$

EEG signals were reconstructed from TRF models as:

$$R_{c}=TRF_{c,b}*S_{c}.$$

**Figure 3.**
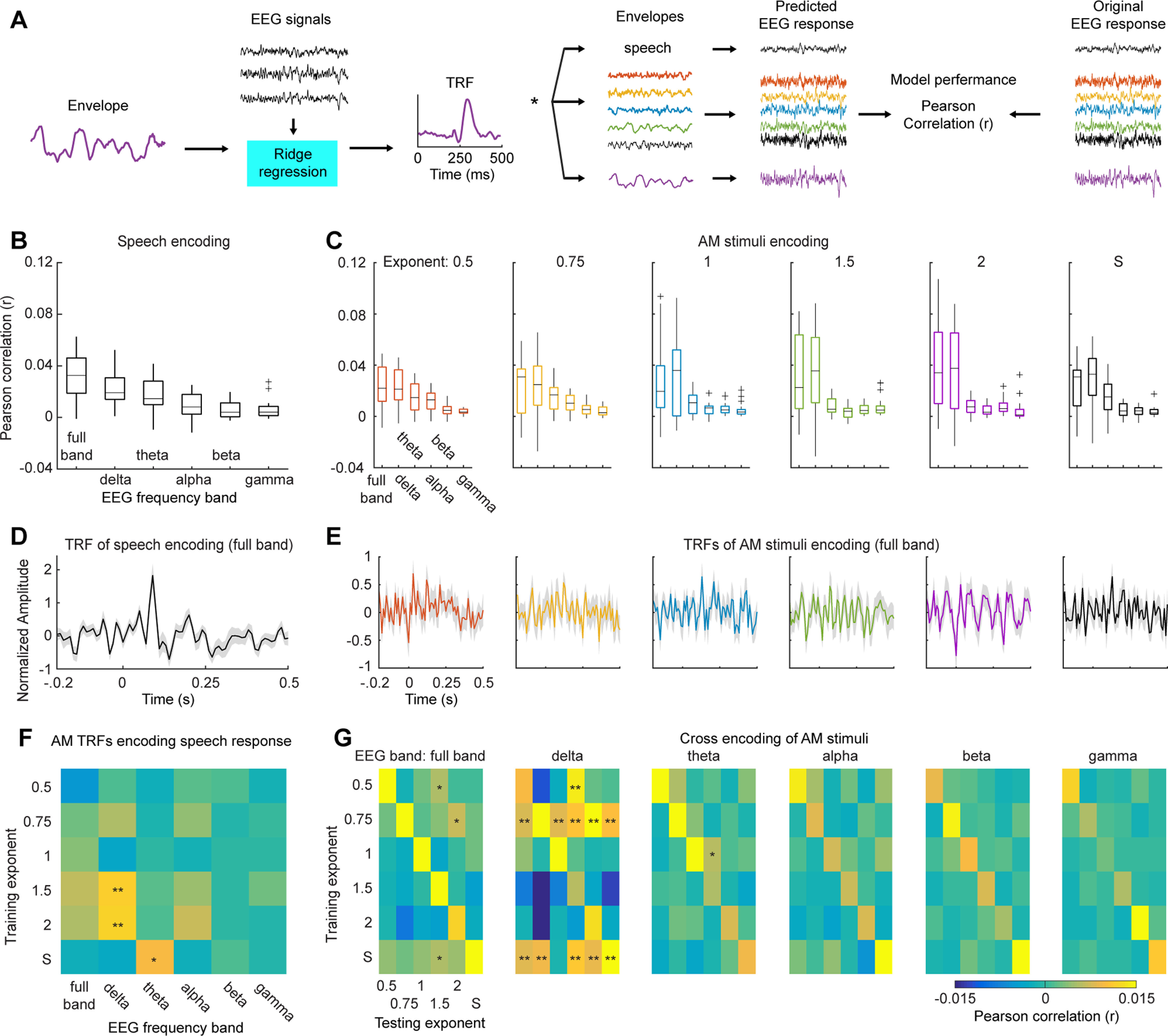
Encoding framework and results. ***A***, Illustration of encoding framework. The AM stimuli of each AM type and the speech material were used to train TRF models. The TRFs from each AM type were then used to predict neural responses to the other AM types and the speech material. ***B***, Box plot of encoding results of speech signals. We trained encoding models using EEG signals of different frequency bands: full band (1–45 Hz), δ (1–3 Hz), θ (4–7 Hz), α (8–12 Hz), β (13–30 Hz), and γ (31–45 Hz). ***C***, Box plots of encoding results of the AM stimuli. From left to right, each panel shows encoding results from the AM stimuli of each AM type. From ***B***, ***C***, it can be seen that the TRFs trained using the full band and the δ and θ bands better predicted neural responses to both the AM stimuli and the speech material. ***D***, TRF for the speech material. ***E***, TRFs for the AM stimuli. The shaded area represents ±1 SE over participants. ***F***, AM TRFs cross-encoding neural responses to speech signals. We used the TRFs trained from the AM stimuli to predict neural responses to the speech material. A permutation test was preformed to determine which the TRF models from the AM stimuli significantly predict speech neural responses (for more details, see Results). We found that the TRFs from the AM stimuli of 1/f modulation spectra of exponent 1.5 and 2 can best predict speech neural responses in the δ band (*p *<* *0.01). ***G***, Cross-encoding between the AM stimuli. We used the TRFs from the AM stimuli of one AM type to encoding neural responses to the other AM types. From left to right, each confusion matrix represents each neural band. The results along the diagonal show the encoding results of one type of AM stimuli with its own TRF model. A permutation test was preformed to determine significant encoding results for each neural band (for more details, see Results); * represents one-sided α level of 0.05; ** represents one-sided α level of 0.01.

The encoding framework included two stages (illustrated in Fig. 3*A*): a training stage to derive TRFs for the AM stimuli and the speech material and to evaluate how well EEG signals can be predicted; a cross-encoding stage to test how TRFs from the AM stimuli predict EEG signals of the speech material and how TRFs from one AM type predicts EEG signals of the other AM types.

At the training stage, we used 50 stimuli of each AM type and their corresponding EEG recordings as a training set to derive TRFs and a leave-one-out validation procedure was conducted to determine the optimal λ that gave the highest encoding performance. The model performance was measured by averaging Pearson correlations (*r*) of all the leave-one-out trials between the EEG recordings and their corresponding predictions under the optimal λ. Correlation coefficients were first transformed using Fisher’s Z-transformation and then averaged. Further analyses were conducted on the transformed coefficients. For the speech material, we divided the speech material and its EEG recording into ten segments. Nine segments were used to derive TRFs and one segment was used as a validation set, and therefore a 10-fold validation procedure was conducted to determine the optimal λ. The Pearson correlations of the ten validations under the optimal λ were averaged and used as the model performance for the speech material.

At the cross-encoding stage, we first applied the derived TRFs and λ values from the AM stimuli to each of the ten segments of the speech material and the EEG recordings. Each predicted EEG response was compared with its original recording, and then the encoding performance was quantified by averaging model performances across the ten segments. Second, we applied the derived TRF from one AM type to the other five AM types. We calculated model performance on each trial of one AM type and then averaged the model performances for 50 trials for each AM type, which was used as the encoding performance for this AM type using another TRF from another AM type. Therefore, a six-by-six cross encoding matrix was created. On the diagonal, the encoding performance was calculated using the TRF from one AM type to apply on the 50 trials of the same AM type, which represented an upper-bound for the cross-encoding performance. TRFs were calculated using the multivariate TRF (mTRF) Toolbox (Crosse et al., 2016).

We tested encoding performance of each frequency band of EEG signals by dividing the EEG signals into five neural bands using a fliterbank of two-pass bandpass Butterworth filters with an order of 4 following conventional definitions: δ (1–3 Hz), θ (4–7 Hz), α (8–12 Hz), β (13–30 Hz), and γ (low γ) bands (31–45 Hz). The encoding procedure described above was conducted in each neural band and in the range between 1 and 45 Hz. The rationale here is that the auditory cortical responses may only encode acoustic dynamics in certain neural bands but not all. Therefore, by decomposing EEG signals into different frequency bands, we could investigate which neural band specifically encodes acoustic dynamics of different AM types.

We further investigated which frequency ranges of modulation spectra of the AM stimuli were best encoded in each neural band. We filtered the amplitude envelopes of the AM stimuli calculated above using a fliterbank of two-pass bandpass Butterworth filters with an order of 2 and decomposed the amplitude envelopes into frequency bands linearly distributed from 1 to 45 Hz with steps of 2 Hz. We repeated the encoding procedures for each modulation band of amplitude envelopes using each neural band of EEG signals.

## Results

### Tone detection performance modulated by the shape of modulation spectra and explained by local SNR

#### Behavioral results

The behavioral results (Fig. 1*E*) demonstrate that participants’ sensitivity to tones (D-prime values) were modulated by the shapes of modulation spectra of the AM stimuli, although the global SNR (−10 dB) was the same across all stimuli. The behavioral performance was examined using a one-way repeated measures ANOVA (rmANOVA) with the main factor of AM Type. We found a significant main effect of AM Type (*F*_(5,90)_ = 2.32, *p *=* *0.025, η_p_^2^ = 0.131). To further examine in which AM type tone detection performance is significantly better than in other AM types, we conducted a permutation test. For each participant, we permutated labels for AM types and randomly assigned D-prime values to different AM types to form a new dataset. The permutated D-prime values were then averaged across the participants and the median was calculated for the each AM type in this new dataset. As the labels for AM types were permutated and the medians of different AM types can be considered to be unspecific to each AM type, we then averaged the derived medians of the new permutated dataset. We repeated this procedure 1000 times and derived a threshold of a one-sided α level of 0.01 (Fig. 1*E*, thin black line). This permutation test avoided the problem of multiple comparison and directly addressed the question that we were interested (Teng and Poeppel, 2020). We found that tone detection performance in the AM type of 1/f modulation spectrum of exponent 2 was significantly higher than in other AM types.

#### Local SNR

As different AM spectra led to different temporal dynamics in the AM stimuli, local SNR may be modulated by AM types. Therefore, we calculated local SNR using rectangular temporal windows of different sizes (for details, see Materials and Methods). We found that the local SNRs of tones in different AM stimuli were indeed modulated by AM spectra and varied with temporal window size (Fig. 1*F*). We then correlated the behavioral results with the local SNRs and found that the correlation coefficients decreased as the temporal window size increased (Fig. 1*G*), which was examined by a one-way rmANOVA with Window size as the main factor (the main effect: *F*_(8,144)_ = 3.92, *p *<* *0.001, η_p_^2^ = 0.179; linear trend: *F*_(1,18)_ = 7.48, *p *=* *0.014, η_p_^2^ = 0.294). The correlation coefficients were first transformed using Fisher’s Z transformation and then went through statistic tests. To further determine whether correlation at each window size was significant above chance, we conducted a permutation test by shuffling labels of AM spectra for local SNRs for each participant at each window size and correlated the shuffled local SNRs with the behavioral results. This procedure created a new averaged correlation coefficient across participants. We repeated this procedure 1000 times and derived a threshold of one-sided α level of 0.01 at each window size (Fig. 1*G*, dashed line). We found that the correlations between the behavioral results and the local SNRs were significant at all the window sizes, with the highest correlation at the smallest window size (30 ms). This result suggests that different AM spectra resulted in different local SNRs, which explained differences of tone detection performance across AM types.

The best tone detection performance was observed for the AM stimuli of 1/f modulation spectrum of exponent 2 probably because the AM stimuli of exponent 2 have more modulation components in the low-frequency range. Although tones were embedded randomly in the AM stimuli, there were more chances for tones to be in a position where local amplitude of the AM stimuli of exponent 2 was low. However, an alternative explanation is that the AM stimuli of exponent 2 fluctuate slowly and hence the acoustic changes are more predictable compared with the other AM stimuli. Listeners can better predict envelope changes and monitor the “surprise” caused by the inserted tones. It would be interesting to separate between predictability and local SNR by controlling one factor while varying the other, but admittedly we could not fully address this question in the current experiment.

### Onset/offset responses and induced power do not significantly differ between different types of AM stimuli

We calculated induced power for each type of AM stimuli to investigate whether different AM types modulated induced power of different neural bands (Fig. 2*B*,*C*). We chose not to calculate evoked power or phase coherence across trials because each AM stimulus had distinct modulation phases. We addressed the issue on how acoustic dynamics in each type of AM stimuli robustly drive phase-locked neural responses in the following encoding analyses.

We first calculated spectrograms of induced power (Fig. 2*B*), which show clear onset and offset responses in the frequencies below 10 Hz. The suppression of power in the beta band (13–30 Hz) can be seen in the spectrograms after the offset of the stimuli, which was probably caused by motor preparation for the button presses. Increased power compared with the baseline (−1 to −0.75 s) in the θ (4–7 Hz) and the α (8–12) bands can be observed across all the AM stimuli. To quantify power changes induced by different types of AM stimuli, we averaged the induced power between 0.5 s and 4.5 s after stimulus onset to avoid influences from onset and offset response. We conducted a one-way rmANOVA at each frequency from 1 to 45 Hz with the factor of AM type and found no significant effects after adjusted false discovery rate (FDR) correction (Benjamini and Hochberg, 1995; Yekutieli and Benjamini, 1999; *p *<* *0.05) although higher induced power responses to the AM stimuli of exponents 1 and 2 can be observed below 10 Hz.

We next examined whether the AM spectra modulated onset and offset responses. We averaged trials from each AM type in the temporal domain (Fig. 2*D*). We conduced one-way rmANOVA at each time point from −200 to 500 ms with the factor of AM type and found no significant effects of AM type after FDR corrections (*p *<* *0.05) for both the onset and offset responses. This is probably because we added ramping windows to the beginning and the end of the AM stimuli, which diminished the influences of modulation spectra. However, interestingly, the temporal profiles of the onset responses differed from the offset responses. While positive peaks were observed around 250 ms in both the onset and offset responses, large negative responses were shown in the offset responses around 100 ms. This observation indicates that, although both the onset and offset responses were evoked by abrupt changes of acoustic energy, the underlying auditory processes are likely different (Kopp-Scheinpflug et al., 2018). The offset responses were possibly further modulated by the button presses in the experiment after offset of stimuli and reflected neural components of predicting processes, as all the AM stimuli had the same length (5 s), participants likely registered the stimulus length and predicted the end of each AM stimulus. Our previous work (Teng et al., 2018) showed that the onset responses were modulated by different frequency modulation spectra although the same ramping windows were added to the stimuli of different 1/f modulation spectra. This interesting difference between the current experiment and Teng et al. (2018) suggests that not only the shape of amplitude envelopes but also spectral details of sounds significantly modulate auditory evoked responses (Oganian and Chang, 2018; Teng et al., 2019).

### Encoding models of AM stimuli show high specificity to different modulation spectra

We trained encoding models for the AM stimuli and the speech material and derived TRFs (Fig. 3*A*). We first quantified how well the encoding model of each type of stimuli can be used to predict neural responses of different neural bands, so that we can validate the method and provide a replication of our previous findings (Teng et al., 2018) and of speech signals (Di Liberto et al., 2015). We then employed the TRFs from the AM stimuli to predict neural responses to the speech material and tested the degree of specificity of TRFs to the corresponding modulation spectra. This aimed to answer our main question, whether the human auditory system is sensitive to the shape of modulation spectrum and whether the shape of modulation spectra can be used to capture canonical neural responses to speech signals.

We followed conventional procedures to train TRFs models using different types of stimuli (AM stimuli of different AM types and the speech material) and to predict neural responses to the stimuli. For the speech material, we replicated previous findings using EEG (Di Liberto et al., 2015) and showed that neural responses to speech signals measured by EEG can be robustly predicted using the encoding model, with the low-frequency neural signals (δ and θ bands) showing the best encoding results (Fig. 3*B*). We conducted a one-way rmANOVA on the encoding results with Neural band as the main factor and found a significant main effect (*F*_(5,85)_ = 14.33, *p *<* *0.001, η_p_^2^ = 0.457) and a significant linear trend (*F*_(1,17)_ = 33.17, *p *<* *0.001, η_p_^2^ = 0.661). The encoding results of the AM stimuli are shown in Figure 3*C*. We conducted a two-way rmANOVA on the prediction performance of the AM stimuli with AM type and Neural band as the main factors. We found a significant effect of Neural band (*F*_(5,90)_ = 47.86, *p *<* *0.001, η_p_^2^ = 0.727) but not of AM type (*F*_(5,90)_ = 0.25, *p *=* *0.940, η_p_^2^ = 0.014). The interaction effect is not significant (*F*_(25,450)_ = 1.21, *p *=* *0.224, η_p_^2^ = 0.063). The linear trend of Neural band is significant (*F*_(1,18)_ = 60.21, *p *<* *0.001, η_p_^2^ = 0.700) and suggests that the full band and the low-frequency bands show better encoding performance than the high-frequency bands. The TRFs of the corresponding encoding models of the full band are shown in Figure 3*D*,*E*.

We next used the TRFs trained from the AM stimuli to predict neural responses to the speech material in different neural bands (Fig. 3*F*). To determine which TRFs from the AM stimuli can robustly predict the speech responses in different neural bands, we employed a permutation test. For each participant, we first shuffled the labels of the prediction performance for different neural bands and different AM TRFs and then derived a group-averaged encoding result. We repeated this procedure 1000 times and derived thresholds of significance of one-sided α levels of 0.05 and 0.01 for each combination of AM TRF and neural band. We found that the TRFs from the AM stimuli of 1/f modulation spectra of exponents 1.5 and 2 can robustly predict the speech neural responses in the δ band compared with other AM TRFs and neural bands (Fig. 3*F*). The TRF from the AM stimuli of speech modulation spectrum in the θ band can also explain the speech neural responses, which is probably because the θ range of the AM stimuli of the speech modulation spectrum contains higher power and preserves crucial features of speech signals (Ding et al., 2017). It has been shown that speech modulation spectra have a 1/f exponent of 1.5 (Singh and Theunissen, 2003), and here, using the AM stimuli of 1/f exponent 1.5, we could predict the speech neural responses in the δ band. This result indeed suggests that the human auditory system is sensitive to the shape of speech modulation spectrum and an artificial sound with a similar modulation spectrum can drive speech-like neural responses.

We also found that the encoding model from the AM stimuli of 1/f exponent 2 significantly predicted the speech neural responses (Fig. 3*F*), so an alternative explanation could be that the AM stimuli of 1/f exponents of 1.5 and 2 have high modulation power in the δ range than the other AM stimuli (Fig. 1*B*) and hence better explained the speech neural responses in the δ range. Therefore, we conducted a one-way rmANOVA on the prediction performance in the δ band with AM Exponent as the main factor and found a significant main effect (*F*_(5,90)_ = 2.41, *p *=* *0.048, η_p_^2^ = 0.115), though with a small effect size. It is worth noting that all the AM stimuli had modulation spectra of a 1/f shape, which means that the modulation power in the δ range was higher than in the other frequency ranges. Therefore, there exist sufficient modulation components in the δ range to derive TRFs of the δ band to explain the speech responses in all the AM stimuli. However, the encoding performance was extremely low for the TRFs from the AM stimuli of 1/f exponents 0.5, 0.75, and 1 in the δ band (group mean: 0.0011, 0.0054, and −0.0033, respectively), which is not fully consistent with this alternative explanation. It is likely that the ratio of modulation power between the low-frequency range and the high-frequency range, but not the absolute magnitude of modulation power in the δ range, is crucial here, the shape of modulation spectrum matters (see Discussion).

We next conducted cross encoding in different neural bands with the AM stimuli, we used the TRF from one type of AM stimuli to predict the neural responses to the other AM stimuli. The reason for this analysis was that we would like to examine the specificity of the encoding models trained from different AM stimuli. If high specificity across different TRF models is observed, the results can further demonstrate that the shape of modulation spectra plays an important role in driving distinct neural responses. The results are shown in Figure 3*G*. In each neural band, we conducted a permutation test to determine which TRFs from the AM stimuli can robust predict the neural responses to the other AM stimuli. For each participant, we first shuffled the labels of the prediction performance for different training exponents and testing exponents, and then derived a group-averaged encoding results in each neural band. We repeated this procedure 1000 times and derived thresholds of significance of one-sided α levels of 0.05 and 0.01 for each combination of training exponents and testing exponents in each neural band. We found that the TRFs trained using the full band and the θ, α, β and γ bands cannot generalize well from one type of AM stimuli to the others, though some small effects are shown (Fig. 3*G*, far-left and middle panels). In contrast, in the δ band more generalizations were observed. Particularly, the TRFs trained using the AM stimuli of 1/f exponent 0.75 and of the speech modulation spectrum can well predict the neural responses to the other AM stimuli. We further investigated this finding in the following analyses, in which we decomposed the AM envelopes into different modulation bands so that we could have a better understanding on what acoustic components in the AM stimuli enabled such generalizations.

### Contributions of each modulation band to encoding performance

Modulation components of different frequencies in the AM stimuli may be differentially extracted by the human auditory system, which was potentially influenced by the shape of modulation spectra. For example, although all the AM stimuli contained considerable modulation components between 1 and 30 Hz, a high ratio of modulation power between the low-frequency range and the high-frequency range may emphasize the low-frequency modulation components (Fig. 1*A*,*B*). This may be the reason why the shape of modulation spectra is crucial to different natural sounds. However, the prominent modulation power in the low-frequency range may bias TRF models trained using the whole modulation spectra and hence the TRF models take into account mainly the frequency ranges with high modulation power without considering each modulation band with equal weight. Hence, we decomposed the amplitude envelopes of the AM stimuli into different modulation bands and trained a TRF model for each modulation band. This procedure normalized the modulation spectra across frequencies and weighed each modulation band equally during training TRF models.

**Figure 4.**
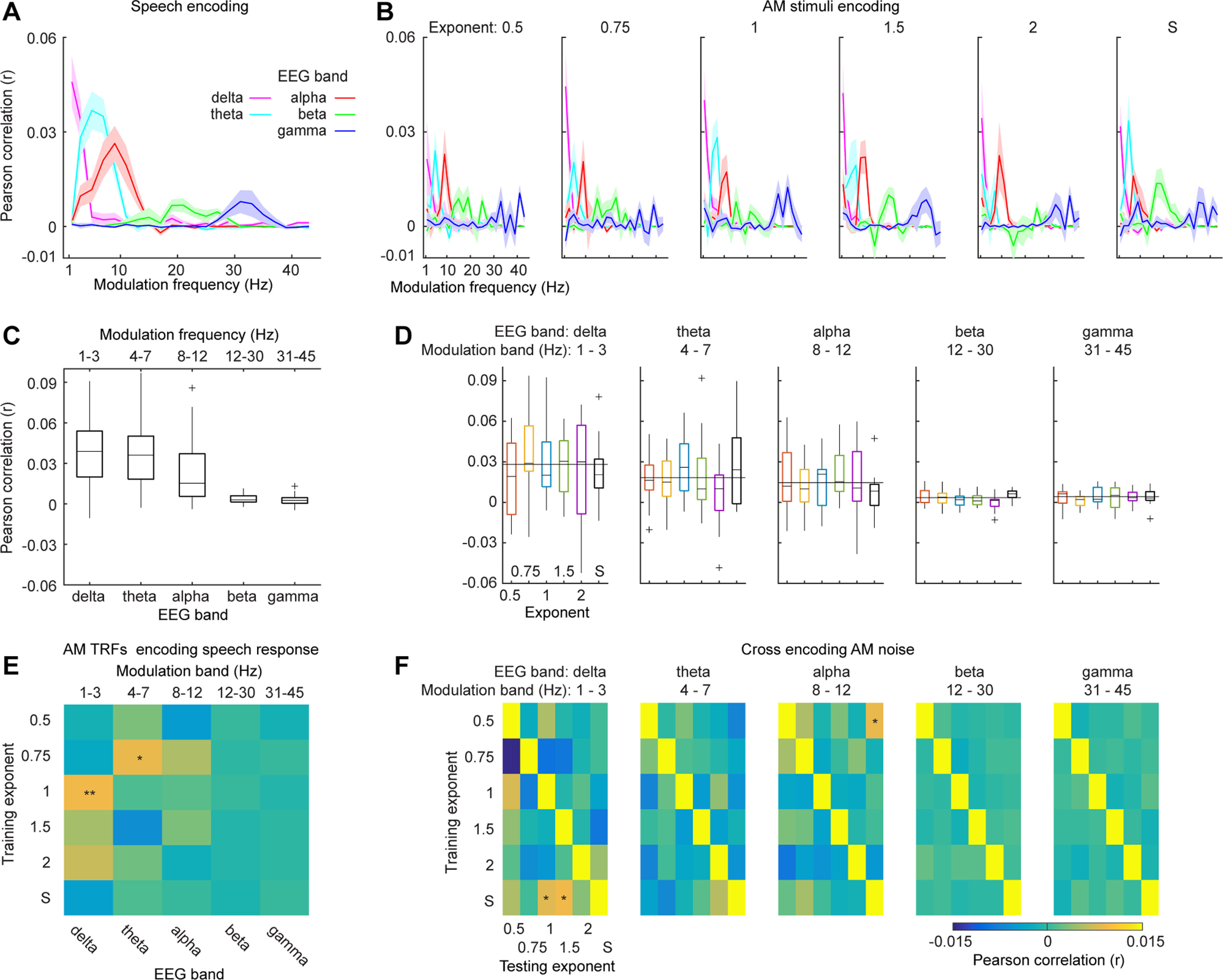
Encoding results of different modulation components. ***A***, Encoding results of modulation components from 1 to 45 Hz for the speech material. The line color codes for different neural frequency bands. ***B***, Encoding results of modulation components from 1 to 45 Hz for the AM stimuli. From the left to right, each panel represents different AM envelope types. It can be seen from ***A***, ***B*** that the neural responses to the speech material and the AM stimuli can be robustly predicted in the low-frequency neural band (<10 Hz) with its corresponding modulation components. ***C***, Box plot of encoding results of speech signals. ***D***, Box plots of encoding results of the AM stimuli. From left to right, each panel shows encoding results of different neural bands. ***E***, AM TRFs encoding neural responses to speech signals. We found that the TRFs from the AM stimuli of 1/f modulation spectra of exponent 1 can best predict speech neural responses in the δ band (*p *<* *0.01). An effect was also shown for the AM stimuli of 1/f exponent 0.75 in the θ band. ***F***, Cross encoding of the AM stimuli. From left to right, each confusion matrix represents each frequency band. The results along the diagonal show the encoding results of one type of AM stimuli with its own TRF model. A permutation test was preformed to determine significant encoding results for each neural band (for more details, see Results); * represents α level of 0.05; ** represents α level of 0.01.

We first calculated encoding results of the speech material and the AM stimuli, respectively, using each modulation component from 1 to 45 Hz with a step of 2 Hz (for details, see Materials and Methods) and plotted the results in Figure 4*A*,*B*. We observed that the neural signals in the low-frequency range (<10 Hz) could be robustly predicted by the encoding models trained using the low-frequency modulation components, which echoes previous findings on robust auditory entrainment in the low-frequency range (Luo and Poeppel, 2007; Lakatos et al., 2008, 2013; Kerlin et al., 2010; Besle et al., 2011; Cogan and Poeppel, 2011; Ding and Simon, 2012; 2013; Kayser et al., 2012, 2015; Henry and Obleser, 2012; Ng et al., 2012; Wang et al., 2012; Herrmann et al., 2013; Peelle et al., 2013; Doelling et al., 2014; Henry et al., 2014; Riecke et al., 2015; Zoefel and VanRullen, 2015). Interestingly, we also observed considerable encoding performance in the high-frequency range, which is consistent with our earlier work on auditory processing in the concurrent θ and γ neural bands (Poeppel, 2003; Boemio et al., 2005; Giraud and Poeppel, 2012; Luo and Poeppel, 2012; Teng et al., 2016, 2017; Teng and Poeppel, 2020).

To further quantify encoding results in each neural band with its corresponding modulation band, we averaged the encoding performance within each neural band and its corresponding modulation band and plotted the results in Figure 4*C*,*D*. For the speech material, we conducted a one-way rmANOVA on the encoding results with frequency band as the main factor and found a significant main effect (*F*_(1,18)_ = 47.95, *p *<* *0.001, η_p_^2^ = 0.727), which suggests that the encoding performance deceased as the frequency ranges increased. For the AM stimuli, we conducted a two-way rmANOVA on the encoding results of the AM stimuli with AM type and frequency band as the main factors. We found a significant main effect of frequency band (*F*_(4,72)_ = 31.24, *p *<* *0.001, η_p_^2^ = 0.634). The main effect of AM type is not significant (*F*_(5,90)_ = 0.95, *p *=* *0.452, η_p_^2^ = 0.050) and the interaction effect is not significant (*F*_(20,360)_ = 1.29, *p *=* *0.184, η_p_^2^ = 0.067).

To further examine which type of AM stimuli was preferably encoded in each frequency band, we conducted a permutation test in each frequency band for different AM stimuli. For each participant, we permutated labels of AM types for the encoding performance to form a new dataset. The permutated encoding results were then averaged across the participants and the median was calculated for each AM type in this new dataset. As the labels for AM types were permutated and the medians of different AM types can be considered to be unspecific to each AM type, we then averaged the medians to derive a value that summarized the medians of this permutated dataset. We repeated this procedure 1000 times and derived a threshold of one-sided α level of 0.01 (Fig. 4*D*, thin black line). In the δ band, we found that the prediction performances of the AM stimuli of 1/f exponents 0.75, 1.5, and 2 were preferably encoded, as well as the AM stimuli of 1/f exponent 1 and of the speech modulation spectrum in the θ band and the AM stimuli of 1/f exponents 1 and 1.5 in the alpha band. These results are consistent with the findings in Teng et al., 2018, showing that in the δ band the stimuli with larger 1/f exponents robustly drive auditory responses and in the θ band the stimuli with 1/f exponent 1 specifically drive auditory responses. Interestingly, the AM stimuli with the speech modulation spectrum also sufficiently drive the θ band auditory response, which is probably because this type of AM stimuli has high modulation components in the θ band and is consistent with previous findings on auditory entrainment of speech signals (Luo and Poeppel, 2007; Peelle et al., 2013; Di Liberto et al., 2015).

We used the TRFs trained from the AM stimuli to predict neural responses to the speech material in different frequency bands (Fig. 4*E*). To determine which TRFs from the AM stimuli can robust predict the speech responses in different frequency bands, we employed the same permutation test in the previous analysis (Fig. 3*F*). We found that the TRFs from the AM stimuli of 1/f modulation spectra of exponent 1 can robustly predict the speech neural responses in the δ band. The TRF from the AM stimuli of 1/f modulation spectra of exponent 0.75 in the θ band can also explain the speech neural responses.

We next conducted cross encoding in different frequency bands with the AM stimuli. The results are shown in Figure 4*F*. We conducted the same permutation test to determine which TRFs from the AM stimuli can robustly predict the neural responses to the other AM stimuli (Fig. 3*G*). We found that the TRFs trained from the AM stimuli with the speech modulation spectrum in the δ can explain neural responses the AM stimuli with 1/f exponents 1 and 1.5. This result well echoes the previous finding that speech signals have a 1/f modulation spectrum of exponent between 1 and 1.5 (Singh and Theunissen, 2003). A generalization from the TRF from the AM stimuli of exponent 0.5 to the neural responses to the AM stimuli with the speech modulation spectrum was also found in the alpha band. However, in general, the TFRs from different AM stimuli cannot be generalized to other AM stimuli.

## Discussion

We generated AM sounds (AM stimuli) with various shapes of long-term modulation spectra to emulate temporal dynamics of natural sounds and investigated how the neural signatures to different modulation spectra can be employed to predict the neural responses to speech signals using an encoding framework. We showed that the neural responses to speech signals can be predicted by the encoding models derived from the modulation spectra similar to the speech modulation spectra in the δ and θ bands (Figs. 3, 4). Moreover, the TRFs derived from the AM stimuli manifested specificity to the corresponding modulation spectra and cannot be well generalized across AM stimuli of different modulation spectra, which demonstrated that the long-term modulation spectrum of sounds indeed drives neural responses characteristic to its specific shape. Furthermore, the long-term modulation spectrum of sounds modulates tone detection performance (Fig. 1) and induced power of neural responses to AM stimuli (Fig. 2).

Much efforts have been devoted to finding canonical receptive fields (e.g., spectral-TRF or TRF) of the auditory system using linear methods (Eggermont et al., 1983; Depireux et al., 2001; Theunissen et al., 2001; Mesgarani et al., 2014), but the estimated auditory receptive fields often depend on the stimuli used and vary with different temporal contexts (Bar-Yosef et al., 2002) and auditory tasks (Fritz et al., 2003). The receptive fields estimated using artificial sounds, such as short tones and spectral or temporal modulated white noise, cannot capture neural processes of complex natural sounds (Laudanski et al., 2012). On the other hand, crucial acoustic dimensions or features in natural sounds (e.g., speech, music, and birdsongs) are not yet clearly understood and therefore the receptive fields estimated from natural sounds suffer from lack of interpretability, what features in natural sounds give rise to such neural responses? One approach to resolve this dilemma is to extract individual features from natural sounds and to investigate each separately, which is the strategy that we employed here. We focused on amplitude envelops of sounds and varied their modulation spectra to investigate how the long-term modulation spectrum of sounds modified neural responses. Indeed, different shapes of the modulation spectra drove TRFs of distinct characteristics, which in general showed high specificity to the corresponding modulation spectra (Fig. 3). This finding revealed that it is key to study neural computations of the auditory system in the temporal domain, the global temporal properties of sounds, characterized by the long-term modulation spectrum, largely modulate neural responses.

Nonetheless, we did observe certain degree of encoding generalizations of TRFs among the AM stimuli and between the AM stimuli and the speech material used (Figs. 3*F*,*G*, 4*E*,*F*). The prominent generalization was found in the δ band. All the stimuli had sufficient modulation power in the δ band range (Fig. 1*B*); the bandwidth of the δ band of both EEG signals and modulation spectra was narrow compared with other frequency bands. There were limited shape variations of modulation spectra in the δ band across different AM stimuli and hence limited variations of temporal dynamics in all the stimuli in the δ band. Therefore, such encoding generalizations in the δ band could be because of the similarity of modulation power and limited temporal variations between the AM stimuli and the speech material. On the other hand, the encoding generalization in the θ band between AM stimuli of speech modulation spectrum and the speech material (Fig. 3*F*) demonstrates that the long-term modulation spectrum of speech signals explains neural responses driven by speech signals, at least under the context of EEG recording. Although the detailed temporal dynamics (controlled by modulation phase) differed between the AM stimuli of speech modulation spectrum and the speech material, the global temporal properties captured by the long-term modulation spectrum sufficed to drive neural responses similar to the ones driven by speech signals recorded by EEG. Therefore, a general conclusion would be that the long-term modulation spectrum of speech signals preserves critical features in speech signals, which drive speech-specific neural responses. However, a conservative conclusion could be that neural responses to speech signals recorded by EEG reflect mainly the responses to broadband envelops of speech signals.

Admittedly, our experimental procedures focused on the acoustic aspect of the speech signals and did not take into account “top-down” processes in speech perception. As the participants could understand the speech materials, top-down or high-level speech processes can largely modulate the neural responses, such as semantic context (Broderick et al., 2018, 2019), listeners’ prior knowledge of speech structure (Teng et al., 2020), and linguistic structure (Kaufeld et al., 2020). It would be worth considering in the future research whether more insights can be revealed using this cross-encoding framework if speech materials of a foreign language, unintelligible to listeners, are used. Consequently, it is worth mentioning that the specificity of speech processing we investigated here mainly involves low-level acoustic processing in the speech processing hierarchy (Hickok and Poeppel, 2007).

One interesting finding of the behavioral results is that, compared with our earlier finding (Teng et al., 2018) in which the temporal window size of around 200 ms was found to best explain tone detection performance in the stimuli with 1/f frequency modulation, here, we found an advantage of small temporal windows (<50 ms; Fig. 1*G*). The discrepancy between two sets of results could be because in the current experiment the stimuli were modulated in the temporal domain (amplitude modulation) whereas in Teng et al., 2018 the stimuli were modulated in the spectral domain. Detecting a tone in the AM stimuli in the current experiment primarily required listeners to monitor fast changes caused by tone onsets in the temporal domain whereas spectral information does not help (amplitudes of all frequency bands were equally modulated); in Teng et al., 2018; listeners had to integrate acoustic information over time to have enough spectral resolution of acoustic signals to separate tones from dynamic spectral backgrounds. This lends support to an interesting hypothesis – to achieve sound recognition, the human auditory system integrates acoustic information over a long temporal window (150–300 ms) to ensure sufficient spectral resolution while employing a short temporal window (<50 ms) to extract fast-changing temporal details (Poeppel, 2003; Boemio et al., 2005; Giraud and Poeppel, 2012; Teng et al., 2016, 2017; Teng and Poeppel, 2020).

In summary, we found high specificity of encoding models to AM sounds with different shapes of long-term modulation spectra. The neural responses to speech signals recorded by EEG can be explained partly by TRFs derived from the amplitude modulated sounds with speech-like modulation spectra. Our results suggest that long-term modulation spectrum is a crucial feature of sounds and that investigating neural processing for different types of long-term modulation spectra can help reveal specialized neural processes of speech perception.

## Synthesis

Reviewing Editor: Christine Portfors, Washington State University Decisions are customarily a result of the Reviewing Editor and the peer reviewers coming together and discussing their recommendations until a consensus is reached. When revisions are invited, a fact-based synthesis statement explaining their decision and outlining what is needed to prepare a revision will be listed below. The following reviewer(s) agreed to reveal their identity: NONE.

The reviewers agree that this manuscript advances the field. Based on the authors’ previous work, the results of the study were somewhat expected but they are still interesting, new and advance the field. Minor comments should be addressed.

Comments:

The authors performed an interesting study aiming to find some canonical temporal functions, which would be critical to speech perception. The authors showed that the neural responses to speech signals are predicted by the encoding models derived from the modulation spectra similar to the speech modulation spectra in the low-frequency delta and theta frequency bands.

The temporal response functions derived from the amplitude modulated stimuli manifested specificity to the corresponding modulation spectra. They also reported that it could not be generalized to the stimuli of different modulation spectra. They argued that it demonstrates the long-term modulation spectrum of sounds drives neural responses characteristic to its specific shape.

I think the study well designed, and the analysis approach and corresponding results are thoroughly described. I feel that the paper already addresses all the required details. I do not have major concerns about the research question and the results.

There are a couple of things to mention, though not major concerns. First, as the authors already described, the main finding somehow partly overlap with their previous results (Teng et al., 2018) as well as Di Lierto et al. (2015). The low-frequency delta and theta results are also somehow expected accordingly. Second, the current findings in relation to speech perception were not discussed with regards to spatial specificity (brain areas) or the interaction with top-down driven modulation effect. It would be good if some thoughts from those perspectives could be added.
